# In-hospital Outcomes and Characteristics of Heart Failure in Sickle Cell Disease

**DOI:** 10.7759/cureus.5660

**Published:** 2019-09-15

**Authors:** Olusayo Fadiran, Abimbola F Balogun, Richard Ogunti, Olajide Buhari, Chandana Lanka, Adebayo Atanda, Daniel A Larbi, Mehrotra Prafulla

**Affiliations:** 1 Internal Medicine, Howard University Hospital, Washington, USA; 2 Internal Medicine, Wake Forest Baptist Medical Center, Winston-Salem, USA; 3 Cardiovascular Disease, George Washington University Hospital, Washington, USA; 4 Cardiovascular Disease, Howard University Hospital, Washington, USA

**Keywords:** heart failure, hospitalizations, african americans, sickle cell disease, outcomes, race/ethnicity

## Abstract

Sickle cell disease (SCD) predominantly affects African-Americans (AAs) in the United States (US). Due to increasing life expectancy in developed countries, SCD-associated cardiomyopathy is typically seen in adults. The aim of this study was to distinguish hospitalization for this phenotype from traditional heart failure (HF) in AAs. We used the National Inpatient Sample (NIS) database to identify HF hospitalizations in AAs between 2005 and 2014 and stratified them according to SCD status. We compared the characteristics and outcomes before and after matching in a 1:3 ratio for age, gender, insurance, smoking status and admission year. Amongst the 1,195,718 HF admissions in AAs, SCD accounted for 7835. The age (mean ± SD) in the SCD cohort was significantly younger (45.66 ± 13.2) vs non-SCD (64.8 ± 15.2), p<0.001. SCD adults had significantly higher rates of pulmonary hypertension (PH), deep vein thrombosis, and pulmonary embolism while non-SCD adults had higher rates of cardiogenic shock and respiratory failure requiring intubation. The national hospitalization rate for HF in AAs increased from 151 to 257 per million between 2005 and 2011 before declining to 241 per million in 2014. There was a decrease in in-hospital mortality in AAs from 4.8% in 2005 to 3.6% in 2014. We also identified independent predictors of in-hospital mortality in SCD with HF. In conclusion, we described hospitalizations for an emerging heart failure phenotype in AAs. Although there is a national decreasing rate of HF hospitalizations in the US, this may not be reflective of the AA population.

## Introduction

Sickle cell disease (SCD) is the most common inherited hemoglobinopathy worldwide. It initially originated from sub-Saharan Africa, Southeast Asia, and India; however, with the growing rate of migration [1], the incidence in developed countries continues to rise, becoming a pandemic [2]. In the United States (US), over 89,000 people live with SCD, amongst whom 90% are African Americans (AA) according to a study using census data obtained by race to estimate the prevalence of SCD [3]. Life expectancy in SCD has improved, particularly in developed countries, resulting in less infant mortality and a growing population of adults living with the disease [3]. Adult complications of SCD represent a major health care burden in developed countries. Cardiovascular (CV) complications are amongst the highest causes of mortality [4]. Earlier emphasis had been on pulmonary hypertension (PH) as the mechanism of morbidity and mortality in this patient population [5-7]. However, there has been a more recent focus on the cardiac phenotype of SCD [8]. Chronic anemia in SCD results in prolonged exposure to a high cardiac output state that causes compensatory eccentric myocardial remodeling, resulting in left ventricular hypertrophy, dilation, and atrial distension [9-11]. This paradigm shift, known as Group 2 PH, as a significant contributor to PH in SCD was confirmed by several studies [12-14].

Significant racial disparities exist in the morbidity and mortality from heart failure (HF). It has been established that HF occurs more in AA at an earlier age and with far worse morbidity, prognosis, and mortality than Caucasians or other ethnicities [15-16]. Explanations for these observed trends have been attributed to poor control of predisposing risk factors such as hypertension, diabetes, obesity, chronic kidney disease, lifestyle habits, socioeconomic status and quality of care [17]. It has been established that diastolic dysfunction is a more common complication than systolic dysfunction in SCD [12,18]. With the recent ethnic approach to HF management, increasing life-expectancy of adults living with SCD and increasing the number of AAs diagnosed with SCD, there is the need to differentiate these two disease phenotypes: AAs with HF and SCD from AAs with HF without SCD in terms of hospitalization, complications, and outcomes. The National Inpatient Sample Database (NIS) is the largest openly available inpatient healthcare database in the US obtained from all states participating in the Healthcare Cost and Utilization Project (HCUP), and it represents more than 97% of the US population [19]. When used with appropriate weighting, the NIS database estimates more than 35 million hospitalizations. It is not insurance-driven, contains de-identified data, and is useful for analyzing specific patient populations [20].

## Materials and methods

We performed a retrospective analysis of HF hospital discharges of AA patients with and without SCD from the National Inpatient Sample (NIS) database and Healthcare Cost and Utilization Project, Agency for Healthcare Research and Quality (HCUP-AHRQ), from the years 2005 to 2014. The NIS is the largest all‐payer database in the US, containing an unweighted estimate of more than seven-million hospital admissions per year. It provides data on demographic characteristics, insurance, length of stay, principal and secondary diagnoses, principal and secondary procedures, comorbidities, and many others. The database contains a weight variable (discharge‐level weight) that allows for the estimation of national trends. Hospitalization involving adult AAs of age ≥18 years and a discharge diagnosis of HF (primary or secondary) were included. Discharges with a diagnosis of HF were identified using the International Classification of Diseases, 9th Revision, Clinical Modification (ICD‐9‐CM) codes. Discharges associated with SCD (primary or secondary) were identified using ICD‐9‐CM codes 282.60, 282.61, 282.62, 282.63, 282.64, 282.68, and 282.69. Records with incomplete data for mortality were excluded.

For each study subject, demographic data and baseline co-morbidities, such as chronic lung disease, diabetes mellitus, hypertension, hypothyroidism, obesity, chronic liver disease, PH, valvular heart disease, atrial fibrillation, chronic kidney disease, end-stage renal disease (ESRD), implantable cardioverter-defibrillator (ICD) in-situ, pacemaker in-situ, history of pulmonary embolism (PE), history of deep vein thrombosis (DVT), long-term anticoagulant use, and smoking status were identified. These co-morbidities were described using the Elixhauser comorbidities in the NIS database as well as ICD DM 9 codes. The Charlson Comorbidity Index (CCI), a validated prognostic tool to predict mortality and to classify prognostic co-morbidities, was calculated for each study subject. A higher CCI is associated with a higher risk of mortality from various large database studies [21]. The primary outcome measure was all-cause in-hospital mortality. Secondary outcome measures include cardiogenic shock, left ventricular assist device (LVAD) utilization, vasopressors utilization, acute respiratory failure, mechanical ventilation, acute kidney injury (AKI), hemodialysis for AKI, acute PE incidence, acute DVT, thrombolytics utilization, and mean length of stay. We matched cases (HF with SCD) with controls (HF without SCD) in a 1:3 ratio for age, gender, insurance, smoking status, and year of admission. We matched based on these variables because of the observed demographic disparity that is mainly driven by the younger SCD population. We used the CCI to describe the comorbidity burden in our study population. Categorical variables were presented as percentages, continuous variables as median±interquartile range, or mean±standard deviation. Comorbidities and clinical outcomes were compared between the SCD group and the non-SCD group in the unmatched and matched cohort using Pearson’s chi-square test for categorical variables. The Wilcoxon‐rank sum and Student t-test were used to compare continuous variables for skewed and normal distribution data where appropriate.

We performed both univariate and multivariable logistic regression analyses to identify independent predictors of in-hospital mortality among the SCD cohort. The multivariable regression analysis included baseline variables that showed a significant univariate association with p-value <0.05. The final model included age as a stratified ordered categorical variable. A trend analysis was performed using appropriate weights (TRENDWT) to compute national estimates. Standardized national rates of HF hospital admission among AAs were calculated for each year (per 1,000,000 AAs) by dividing the estimated number of admissions by the US AA population of adults ≥18 years of age for that year. National population estimates for AAs were obtained for each year from the US Census Bureau, and annual rates were age and sex standardized to the 2014 population. In‐hospital mortality for each year was calculated as the number of deaths per 1000 HF admissions for each year. Data analysis was performed with Stata software (StataCorp LP, College Station, TX). All statistical tests were two‐sided, and tests with p<.05 were considered significant. This study was exempted by the Howard University Hospital IRB, as it does not include identified Health Insurance Portability and Accountability Act (HIPAA) personal information, and the database used is publicly available.

## Results

**Figure 1:**
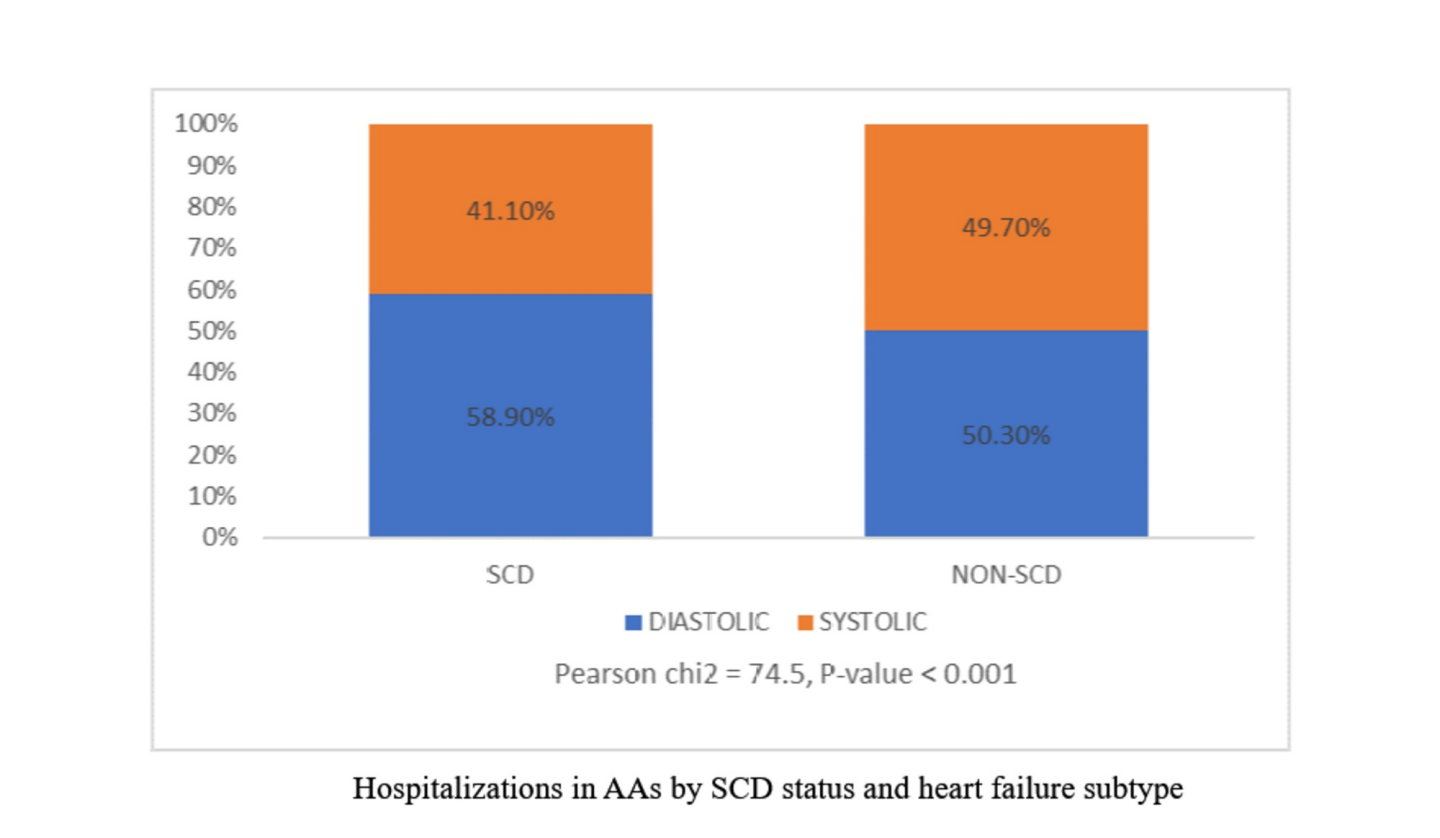
Total Number of Heart Failure Hospitalizations in African Americans by Sickle Cell Disease and Heart Failure Type (HCUP-NIS database: 2005-2014) SCD=Sickle Cell Disease; AA= African American; Total number (n) of heart failure hospitalizations in AAs with distinctive ICD-9-CM codes for systolic and diastolic heart failure were 469,957. Total n of SCD was 2502

HF in SCD(n) accounted for about 0.66% of the 1,195,718 hospital admission for HF in AAs, n=7835. Table 1 shows the baseline characteristics, insurance status, and co-morbidities of both unmatched and matched cohorts. The average age of the SCD group was 45.66 ± 13.2 years as compared with 64.8 ± 15.2 years in the non-SCD group. There was a higher percentage of females in both cohorts. Amongst those with distinct ICD‐9‐CM codes of diastolic and systolic HF (Figure 1), SCD patients had a significantly higher proportion of diastolic heart failure hospital admissions compared with non-SCD AAs. In terms of patient characteristics, other differences between SCD and non-SCD cohorts noted were: non-SCD patients were more likely to have chronic lung disease, CV risk factors like hypertension, diabetes, and obesity as comorbidities, and required cardiac implants, such as pacemakers and defibrillators, in both matched and unmatched studies (p<0.001). SCD patients were more likely to require long-term anticoagulation and have PH, chronic liver disease, PEs, and DVTs in the past (p<0.001).

**Table 1:** Baseline Characteristics of Participants: Pre- and Post-Match Cohorts *Control matching based on age, gender, insurance, current smoking status, and year of admission SCD=Sickle Cell Disease; ESRD=End-Stage Renal Disease; ICD=Implantable Cardioverter Defibrillator; PE=Pulmonary Embolism; DVT=Deep vein thrombosis; IQR=Interquartile Range

Variables	Pre-Match: Unmatched Cohorts			Post-Match: Matched Cohorts*		
	SCD (n =7835)	Non- SCD (n =1,187,883)	p-value	SCD (n = 7638)	Non- SCD* (n =22,914)	p-value
*Age	45.66 ± 13.2	64.8 ± 15.2	<0.001	45.6 ± 13.2	45.6 ± 13.2	0.824
*Gender-female	56.8%	54.7%	0.003	57.1%	57.5%	0.552
*Insurance; Medicare; Medicaid; Private Insurance; Self-Pay	50.6%; 29.3%; 14.1%; 3.6%	64.4%; 15.5%; 12.9%; 4.4%	0.001	50.7%; 29.5%; 13.9%; 3.7%	50.5%; 29.8%; 14.0%; 3.6%	0.706
Chronic Lung Disease	23.1%	30.4%	0.001	23.0%	26.9%	0.001
Diabetes Mellitus	13.6%	47.0%	<0.001	13.6%	40.9%	0.001
Hypertension	50.5%	72.2%	0.001	50.2%	68.6%	0.001
Hypothyroidism	5.6%	7.9%	0.001	5.6%	5.9%	0.226
Obesity	7.6%	18.9%	0.001	7.6%	27.5%	0.001
Chronic Liver Disease	7.5%	3.4%	0.001	7.2%	3.6%	0.001
Pulmonary Hypertension	22.6%	5.5%	0.001	22.4%	6.0%	<0.001
Valvular Heart Disease	9.3 %	6.2%	0.001	9.1%	5.8%	0.001
Atrial Fibrillation	12.9%	20.3 %	0.001	12.9%	11.3%	0.006
Chronic Kidney Disease	20.4%	27%	0.0001	20.4%	20.0%	0.357
ESRD	14.9%	15.4%	0.1486	14.6%	24.1%	0.001
ICD in-situ	3.2%	7.7%	0.001	2.9%	9.3%	0.001
Pacemaker in-situ	1.8%	4.9%	0.001	1.6%	2.4%	0.001
History of PE	3.9%	0.98%	0.001	3.9%	1.5%	0.001
History of DVT	12.3%	4.5%	0.001	12.3%	5.3%	0.001
Long-Term Anticoagulant	8.8%	6.9%	0.001	8.8%	6.6%	0.001
*Current Smoking	6.0%	10.5%	0.001	6.0%	5.7%	0.348
Charlson Comorbidity Index (IQR)	2(1-3)	2(2-3)	0.001	2 (1-3)	2 (1-3)	0.060

**Figure 2:**
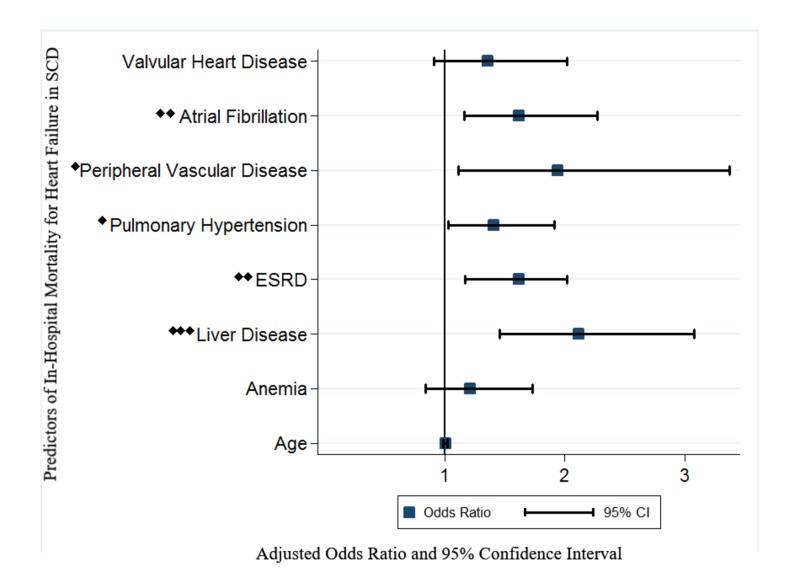
Multivariable Predictors of In-Hospital Mortality of Heart Failure Hospitalizations in Sickle Cell Disease Indicates level of significance: ♦ <0.05 ♦♦ ≤0.01 ♦♦♦ ≤0.001 ESRD=End Stage Renal Disease. Variables included in the model: Significant univariate predictors included in the multivariate model were: age, anemia, chronic liver disease, ESRD, pulmonary hypertension, peripheral vascular disease, atrial fibrillation, and valvular heart disease

**Figure 3:**
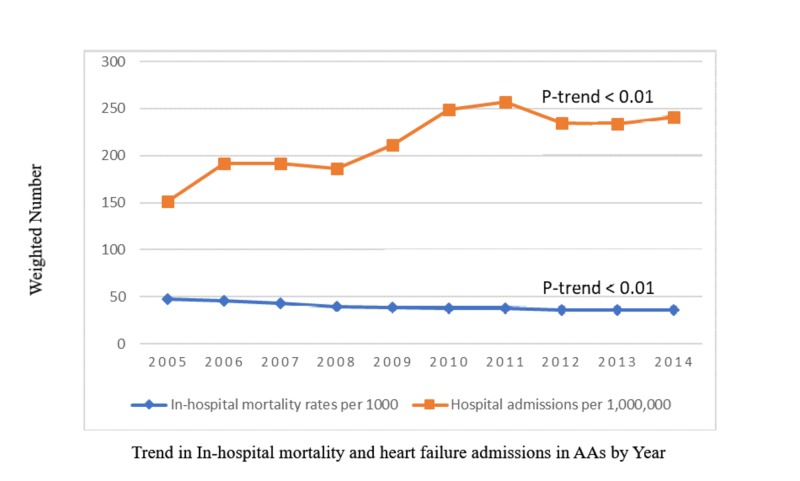
National Weighted Trend of In-Hospital Mortality and Hospital Admissions for Heart Failure in African-Americans over 10 years AA=African-American All-cause In-hospital mortality rates from heart failure among African-Americans per 1000. Heart failure hospital admission per 1000000 African-Americans.

Table 2 highlights the differences in HF hospitalization outcomes between SCD and non-SCD AAs. Cardiogenic shock and use of LVAD for HF management occurred more often in the non-SCD group (p<0.005). Acute respiratory failure and mechanical ventilation were more common complications of HF in non-SCD patients (p<0.001). Acute DVTs and PEs were more common in SCD patients (p<0.05) although after matching, the difference in PE outcome was not significant (p=0.109). Hospital length of stay (mean± SD) for HF was significantly longer in the SCD group than the non-SCD group in both unmatched (7.6 ± 8.2) vs (6.6 ± 8.2) and matched (7.5 ± 8.2) vs (6.2 ± 8.6) cohorts (p=0.001). Overall in-patient mortality of hospitalization for HF was lower in the SCD group: 2.86% vs 3.92%; p=0.001. After matching, the difference in mortality rate was not significant (p=0.298). In the unadjusted analysis to identify predictors of in-hospital mortality in SCD with HF, significant covariates associated with mortality were found, as listed in Table 3. After multivariable adjustment of significant univariate predictors (Figure 2), only ESRD (OR, 1.63; 95% CI 1.17-2.26), atrial fibrillation (OR, 1.62; 95% CI 1.16-2.28), peripheral vascular disease (OR, 1.94; 95% CI 1.12-3.37), PH(OR, 1.41;95% CI 1.03-1.91), and chronic liver disease (OR, 2.13; 95% CI 1.47-3.09) remained significant predictors for mortality in HF hospitalization with SCD. The 10-year weighted trend of admissions and in-hospital mortality for heart failure in AAs (Figure 3) shows a 70% steady increase in hospitalization rate from 2005 (151 per million) to 2011 (257 per million) before declining to 241 per million AAs in 2014. All-cause mortality of heart failure hospitalizations in AAs slowly declined from 4.8% in 2005 to 3.6% in 2014.

**Table 2:** Outcomes of Heart Failure Hospitalizations in SCD and Non-SCD: Pre- and Post-Match Cohorts *Control matching based on age, gender, insurance, current smoking status and year of admission SCD=Sickle Cell Disease; LVAD=Left Ventricular Assist Device; AKI=Acute Kidney Injury; PE=Pulmonary Embolism; DVT=Deep Vein Thrombosis

Outcomes	Pre-Match Cohorts			Post-Match* Cohorts		
	SCD (n = 7835)	Non- SCD (n =1,187,883)	p-value	SCD (n = 7638)	Non- SCD (n =22,914)	p-value
Cardiogenic shock	0.42%	1.04%	0.003	0.42%	1.30%	0.001
Use of vasopressors	0.40%	0.52%	0.152	0.40%	0.57%	0.077
LVAD use	0.05%	0.44%	0.01	0.05%	0.60%	0.001
Acute respiratory failure	7.4%	9.4%	0.001	7.4%	8.6%	0.001
Mechanical ventilation	5.0%	6.9%	0.001	5.1%	6.6%	0.001
Acute kidney injury	20.5%	21.8%	0.008	20.5%	17.6%	0.001
Hemodialysis for AKI	11.2%	8.9%	0.001	11.2%	12.2%	0.296
Acute PE	1.8%	1.3%	0.001	1.8%	1.5%	0.109
Acute DVT	2.2%	1.8%	0.026	2.2%	1.5%	0.001
Thrombolytics utilization	0.41%	0.40%	0.837	0.41%	0.38%	0.672
Length of stay (mean± SD)	7.6 ± 8.2	6.6 ± 8.2	0.001	7.5 ± 8.2	6.2 ± 8.6	0.001
In-patient mortality	2.86%	3.92%	0.001	2.86%	2.64%	0.298

**Table 3:** Univariate Association of In-Hospital Mortality in Sickle Cell Disease Hospitalizations for Heart Failure * indicates significance p<0.05 ESRD=end-stage renal disease, ICD=implantable cardioverter defibrillator, Hx=history

Variables	Odds Ratio (OR)	95% confidence intervals (CI)	p-value
Age (yrs)			
18 – 35	1.00	ref.	-
35 – 45	1.10	0.72 – 1.70	0.645
45 – 55	1.26	0.84 – 1.88	0.257
* > 55	1.74	1.17 – 2.58	0.006
Gender-female	0.86	0.66 – 1.12	0.256
Insurance	0.87	0.76 – 1.01	0.070
Diabetes Mellitus	0.95	0.63 – 1.40	0.790
Hypertension	0.96	0.74 – 1.26	0.780
Hypothyroidism	1.22	0.72 – 2.09	0.450
*Peripheral Vascular Disease	2.69	1.59 – 4.56	<0.001
*Chronic Liver Disease	2.52	1.75 – 3.62	<0.001
*Pulmonary Hypertension	1.42	1.05 – 1.90	0.019
*Heart Valvular Disease	1.69	1.12 – 2.41	0.010
*Atrial Fibrillation	2.07	1.50 – 2.86	<0.001
Long-Term Anticoagulation	1.01	0.64 – 1.62	0.944
Chronic Kidney Disease	0.89	0.63 – 1.25	0.518
*ESRD	1.95	1.43 – 2.67	<0.001
*Anemia	1.58	1.12 – 2.22	0.008
ICD in-situ	1.09	0.51 – 2.34	0.824
Pacemaker in-situ	0.72	0.23 – 2.29	0.583
Hx of Alcohol Use Disorder	1.17	0.48 – 2.89	0.726
Smoking	1.21	0.71 – 2.03	0.461

## Discussion

Using an extensive nationwide database, we have demonstrated that respiratory complications, hemodynamic cardiac complications, and the use of implantable or pump assist devices are significantly lower in SCD AAs as compared to non-SCD AAs. An explanation for this finding could include less frequent use of these devices due to a lack of clinical evidence to support its efficacy in the SCD population. Another reason could be a higher preponderance of diastolic heart failure in SCD as was shown in our study. This relatively higher predominance of diastolic dysfunction in SCD has been confirmed in several studies [22-23]. Unlike traditional AAs with hypertension and aging as mechanisms for diastolic dysfunction, the mechanism of diastolic dysfunction in SCD is primarily a result of eccentric remodeling resulting from a high-output anemic state and microvascular obstruction, both at the level of the myocardium and peripheral organs [24]. The increased use of ICDs for heart failure management in non-SCD AAs reflects a more appropriate indication for their use in this disease phenotype.

Our study also confirms that adults with SCD in developed countries are living up to the seventh decade although this is relatively lower than life expectancy in non-SCD adults. The mean age of SCD hospitalizations for HF in our study is higher than hospitalizations or admissions for vaso-occlusive crises (VOC). In one study, the mean age for VOC was 31.7 years [25]. Our study confirms that HF may be an age-related complication of SCD now evident because of increasing survival. Our study indicates a significantly higher prevalence of PH in SCD heart failure hospitalizations. The higher prevalence of PH in SCD-AAs is most likely due to a combination of both precapillary and postcapillary pulmonary hypertension as contributors. Unlike PH in traditional non-SCD AAs, which is a result of increased pulmonary venous pressure from left-sided heart failure (World Health Organization (WHO) group 2 classification system), PH in SCD (WHO group 5 classification system) is multifactorial, resulting from high-flow cardiac output, increased left-sided venous pressure, nitric oxide scavenging by cell-free hemoglobin, chronic hypoxemia induced by repeated acute chest crises or pulmonary microinfarcts, and chronic thromboembolism amongst others [26]. This analysis reveals that SCD patients with heart failure hospitalizations have a higher prevalence and complication of venous thromboembolic events consistent with other studies demonstrating an increased incidence of DVTs and PEs in SCD [27].

Several studies have confirmed hypertension, diabetes, obesity, smoking, and coronary artery disease (CAD) as risk factors for developing heart failure. Our study shows a greater frequency and pervasiveness of these HF risk factors in traditional non-SCD AAs as was shown by Ogunbayo et al. in SCD patients with myocardial infarction [28]. From our study, the national admission rate for HF in AAs increased over the years with a more recent decline in the later years. This finding is similar to the study by Ziaeian et al., which did not reveal a significant change over the years in hospitalization for HF in the AA ethnic subgroup despite an overall national decrease in hospitalization for HF [29]. Other observational studies involving community cohorts have also shown a higher rate of rehospitalizations for HF in AAs and a worse burden as compared with other ethnicities [30]. This trend observed in our study and others reveals the underlying socioeconomic determinants of health affecting AAs. With inadequate access to ambulatory specialist care and low medication compliance rates to prevent decompensation, HF hospitalization rates in AAs are likely to escalate. More importantly, adequate control of predisposing risk factors such as hypertension amongst others is a daunting challenge in this ethnic population. We identified that in-hospital mortality of HF in SCD was slightly lower than in non-SCD patients. The increased mortality in traditional non-SCD patients may be due to the increased age of hospitalization for HF and a higher comorbidity burden as reflected in the CCI score, although after matching, these differences were not obvious. Mortality statistics for SCD are mostly population-based longitudinal studies or invasive hemodynamic investigational studies and frequently identify PH as a major predictor [4,6]. Although many studies have identified predictors of mortality and sudden cardiac death in SCD, there is a paucity of studies focusing on in-hospital mortality for left-sided HF in this population.

Our study shows that PH raises the odds of mortality for HF admissions in SCD independent of age, anemia, ESRD and other comorbidities. One of the significant predictors of mortality for HF hospitalizations in SCD in our study was chronic liver disease. Chronic liver disease was significantly more prevalent in the SCD cohort. Chronic liver disease is complex and could result from the cor-pulmonale complication of PH in SCD in addition to multiple other factors, including intrahepatic sickling, iron overload secondary to hemotransfusion, and gallstone disease. Another independent predictor of mortality of HF hospitalizations in SCD was ESRD, which is also an independent predictor of mortality for HF hospitalizations in the general population.

Several limitations exist in our study. This is a cross-sectional study that contains de-identified data, hence follow-up data and readmissions or rehospitalization information cannot be ascertained in this study. Even though the ICD-9 diagnostic codes have been validated with a highly acceptable level of accuracy for HF hospitalizations, their use for distinguishing diastolic from systolic HF has not been well studied. We included both primary and secondary diagnosis of HF in our study to increase our sensitivity. Including secondary diagnosis for HF in discharge diagnosis codes has been shown to decrease the accuracy with studies reporting positive predictive values consistently less than 90%. Furthermore, outcomes assessment is limited to the in-patient setting and post-discharge care and events are missing. We did not have access to the diagnostic method for heart failure in this large study, as such an HF diagnosis was not standardized across this large national representative sample. Moreover, the criteria for diagnosing HF in SCD has not been fully evaluated and validated. We could not account for race/ethnic misclassification in this study. We did not have access to the severity of presentation, medications, and interventions done for each presentation. Stratifying for HF severity and intensive care admissions could have influenced our results in such a way that we could better understand the reasons behind the disparities.

## Conclusions

Despite all these limitations, we were able to identify a distinct phenotype of HF predominantly in AAs due to the increasing life-expectancy in SCD that might require a different approach to therapy. While the national trend for HF hospitalizations in the US has been on a decline, this may not be reflective in the AA population of the US. This sheds more light on the need for race stratification when embarking upon strategies to investigate and relieve the burden of HF. More importantly, we were able to identify specific predictors of in-hospital mortality for HF in this emerging population of adults living with SCD.
